# Inflammatory and immune pathways in Ménière’s disease: a multi-database bibliometric and visualized analysis of research trends

**DOI:** 10.3389/fcimb.2026.1951199

**Published:** 2026-09-29

**Authors:** Yingfu Zhou, Jiahui Xiong, Yong Huang, Qinxiu Zhang

**Affiliations:** 1 Department of Otorhinolaryngology, The Second Affiliated Hospital of Chengdu University of Traditional Chinese Medicine, Chengdu, China; 2 School of Clinical Medicine, Chengdu University of Traditional Chinese Medicine, Chengdu, China; 3 Department of Otorhinolaryngology, Hospital of Chengdu University of Traditional Chinese Medicine, Chengdu, China

**Keywords:** bibliometric analysis, immune dysregulation, inflammation, Ménière’s disease, visualization

## Abstract

**Background:**

Ménière’s disease (MD) is a chronic inner ear disorder characterized by recurrent episodes of vertigo, fluctuating sensorineural hearing loss, tinnitus, and aural fullness. Growing evidence indicates that aberrant immune regulation, inflammation, and allergic reactions are involved in the development of MD. However, a systematic synthesis of the current research landscape and evolving hotspots in this field remains lacking.

**Methods:**

Publications concerning immune-inflammatory mechanisms in MD published between 1 January 2006 and 31 December 2025 were retrieved from the Web of Science Core Collection and Scopus databases. Bibliometric and visual analyses were performed using R-bibliometrix, VOSviewer, CiteSpace, and Python.

**Results:**

A total of 868 publications were included in this study, with annual output showing a sustained upward trend. The United States, China, and Italy were the major contributing countries. Core journals and research teams in this field have been predominantly concentrated in the areas of otology, neuro-otology, and vestibular medicine. Research hotspots have progressively shifted from clinical phenotypes, endolymphatic hydrops, and local therapeutic interventions toward immune-inflammatory mechanisms, encompassing cytokine networks, innate immune signaling, allergy-related pathways, and imaging-based phenotypes. Among these, the IL-1β/TNF-α axis, the NF-κB/TLR signaling pathway, and IgE-mediated immune responses have emerged as prominent research directions.

**Conclusions:**

This study systematically delineates the global research landscape, knowledge structure, and evolving hotspots in the field of immune-inflammatory research in MD. The findings highlight major research themes and emerging trends, providing a comprehensive research landscape that may help prioritize future investigations into disease mechanisms, biomarker validation, patient stratification, and immune-targeted therapeutic approaches in MD.

## 1 Introduction

Ménière’s disease (MD) is a chronic disorder of the inner ear characterized by recurrent episodes of vertigo, fluctuating sensorineural hearing loss, tinnitus, and aural fullness. Histopathologically, the disease is typified by endolymphatic hydrops, which can substantially impair patients’ quality of life (Merchant et al., 2005; Ahmad and Lin, 2023). Although the precise pathophysiological mechanisms underlying MD remain incompletely understood, accumulating evidence suggests that immune-mediated inflammatory responses play a pivotal role in its onset and progression (Yuan et al., 2025). Elevated levels of pro-inflammatory cytokines, aberrant autoantibody expression, and immune cell infiltration have been observed in the endolymphatic sac and vestibular organs of patients with MD, suggesting that dysregulated immune responses may contribute to inner-ear homeostatic disturbances and vestibular–cochlear dysfunction (Larsen et al., 2015; Lopez-Escamez and Perez-Carpena, 2024; Xie et al., 2024).

In addition to abnormalities in pro-inflammatory mediators, including TNF-α, IFN-γ, IL-6, and IL-1β, genetic studies have implicated immune-related signaling pathways, particularly NF-κB and TLR signaling, in MD susceptibility and clinical heterogeneity (Huang et al., 2022; Jia et al., 2023; Monaghan et al., 2025; Cruz-Granados et al., 2026). More recently, DNA methylation, transcriptomic, and single-cell multi-omic studies have identified distinct molecular and immune profiles among patients with MD, suggesting that immune-inflammatory alterations may characterize specific molecular subgroups rather than represent a uniform feature across all patients (Flook et al., 2021; Cruz-Granados et al., 2024). These findings support an emerging view in which genetic and epigenetic factors, immune regulation, and inner-ear microenvironmental changes may interact to shape the diverse clinical manifestations of MD.

Over the past two decades, research on the immune-inflammatory aspects of MD has expanded rapidly, encompassing clinical phenotypes, cytokine networks, genetic susceptibility, and immune regulatory mechanisms (Wu et al., 2026). Nevertheless, the existing literature remains fragmented, and conventional narrative reviews are insufficient to provide a systematic overview of the knowledge structure, research evolution, and emerging trends in this field. Therefore, this study integrated publications from the Web of Science Core Collection (WOSCC) and Scopus databases (2006–2025) and applied bibliometric and visualization approaches to characterize the global research landscape and thematic evolution of immune-inflammatory research in MD. The findings are expected to provide a structured overview of the research landscape and help identify emerging areas for future investigation, including biomarker validation, patient stratification, and immune-targeted therapeutic research.

## 2 Materials and methods

### 2.1 Data sources and preprocessing

Publications included in this study were retrieved from two major academic databases, the WOSCC and Scopus, to enhance the comprehensiveness and robustness of the analysis. The WOSCC and Scopus are currently the most widely used databases in bibliometric research, offering broad coverage, highly standardized indexing, and comprehensive data structures. Therefore, these two databases were selected as the data sources for the present study. In contrast, databases such as PubMed do not provide complete cited-reference fields and thus do not adequately meet the analytical requirements of this study. The search period spanned from 1 January 2006 to 31 December 2025. Only publications written in English and classified as either Article or Review were included.

The search strategy was constructed using Boolean operators by combining terms related to Ménière’s disease (MD) with immune- and inflammation-associated keywords. Specifically, the WOSCC search was performed using the Topic Search (TS) field, whereas the Scopus search was conducted using the TITLE-ABS-KEY field. The detailed search strategies for both databases are provided in **Supplementary Table 1**.

The initial search yielded 422 records from WOSCC and 804 records from Scopus. Data integration and deduplication were performed according to standardized procedures commonly adopted in bibliometric studies (Fei et al., 2025; Yan et al., 2025; Sui and Yin, 2026; Wang et al., 2026). Specifically, we first exported the full records from the WoSCC and Scopus databases and harmonized the data fields and formats. The datasets from the two databases were then integrated using the R package Bibliometrix in combination with custom Python scripts. Duplicate records were identified using a hierarchical matching strategy. DOI was used as the primary matching criterion, and records with available DOIs were cross-checked accordingly. For records with missing or inconsistent DOI information, standardized article titles, author information, and publication years were further compared. To account for differences in author-name formats between the databases, author information was standardized by harmonizing capitalization and removing redundant spaces and punctuation. Article titles were similarly standardized by harmonizing capitalization and removing redundant spaces and punctuation that did not affect record identification. For records with missing DOIs or minor discrepancies in titles between databases, further verification was performed based on a combination of title, author information, and publication year. Records that could not be reliably resolved through automated matching were manually reviewed to minimize both erroneous removal and inadvertent retention of duplicate records. Ultimately, 868 unique publications were included for further analysis, comprising 618 original articles and 250 review articles (**Figure 1**). The titles, abstracts, and author keywords of all included publications were extracted to establish the corpus for subsequent analyses.

**Figure 1:**
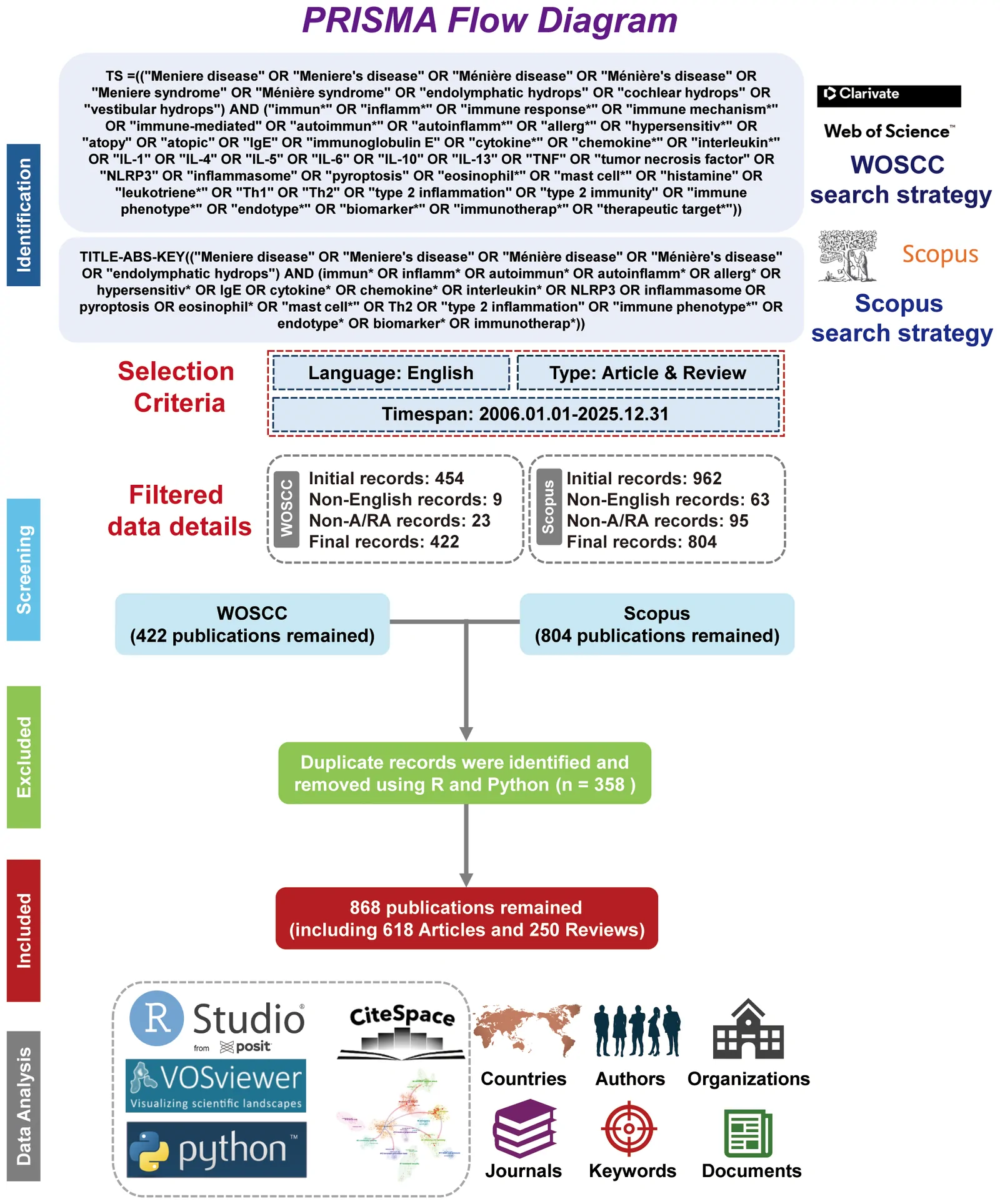
PRISMA flow diagram illustrating the literature screening and selection process. The search strategy used keywords related to Ménière’s disease and immunology across the Web of Science Core Collection (WOSCC) and Scopus databases. A total of 454 records from WOSCC and 962 from Scopus were initially retrieved, restricted to English-language articles and reviews published between January 1, 2006, and December 31, 2025. After removing non-article/review document types, 422 WOSCC records and 804 Scopus records remained. Following duplicate removal (n = 358), 868 unique publications (618 articles and 250 reviews) were included in the final analysis. Data visualization and network analyses were performed using R, Python, VOSviewer, and CiteSpace.

### 2.2 Bibliometric analysis

A combination of bibliometric and visualization tools was employed to comprehensively analyze the included publications. Descriptive bibliometric analyses were conducted using the R-bibliometrix package (v5.0) implemented in R (v4.4.5), including annual publication trends, citation characteristics, leading countries, authors, journals, and citation analyses. In addition, the package was used to extract network data and generate thematic evolution bubble maps to visualize changes in research themes over time (Arruda et al., 2022). Trend Topics analysis in R-bibliometrix was performed using the top 120 most frequent terms. The Louvain algorithm was applied for thematic clustering with a minimum frequency threshold of 5 occurrences per thousand publications, and two representative labels were displayed for each thematic cluster. Keyword clustering analysis was further performed using CiteSpace (v6.2.R1). The time slice was set to one year, and network construction followed the parameters: g-index (k = 25), LRF = 2.5, L/N = 10, LBY = 5, and e = 1.0. Keyword burst detection and reference burst analyses were conducted with a minimum duration of two years, whereas the remaining parameters were retained at their default settings (α = 2.0 and γ = 1.0) (Synnestvedt et al., 2005). In addition, VOSviewer (v1.6.20) was used to construct author citation networks, with thresholds set at a minimum of seven publications and zero citations (Arruda et al., 2022). The raw data generated by the aforementioned software were subsequently imported into the Python environment (v3.14), where additional visualizations were produced using the ‘pandas’, ‘matplotlib’, ‘seaborn’, and ‘networkx’ libraries.

## 3 Results

### 3.1 Publication characteristics

Between 2006 and 2025, 422 and 804 publications meeting the inclusion criteria were retrieved from WOSCC and Scopus, respectively. After data integration and deduplication, a total of 868 unique publications from 397 sources were included in the final analysis, comprising 618 original articles and 250 review articles (**Table 1**).

**Table 1:** Overall comparison of the datasets across databases.

Description	WOSCC	Scopus	WOSCC+Scopus
Timespan	2006:2025	2006:2025	2006:2025
Sources (Journals, Books, etc)	168	370	397
Documents	422	804	868
Annual Growth Rate %	13.45	8.17	8.51
Document Average Age	7.91	7.86	7.95
Average citations per doc	22.19	23.33	23.22
References	14031	82288	61945
Keywords Plus (ID)	1242	8964	8355
Author’s Keywords (DE)	1051	1958	2089
Authors	1944	3432	3916
Authors of single-authored docs	15	40	42
Single-authored docs	15	40	42
Co-Authors per Doc	6.13	5.62	5.58
International co-authorships %	25.83	20.15	20.28
article	333	561	618
review	89	243	250

The most direct way to evaluate the development of a research field is to examine its annual publication output. The annual number of publications exhibited a steady upward trend in both WOSCC and Scopus throughout the study period (**Figures 2A–D**). Overall, the compound annual growth rate (CAGR) reached 8.51%, and the average number of citations per document was 23.22. The most pronounced increase in publication output occurred between 2019 and 2022, during which the annual growth rate reached 97.56%, substantially exceeding the overall growth trend (**Figure 2E**). With respect to collaboration patterns, the average number of authors per publication was 5.58, and 20.28% of publications involved international collaboration (**Figure 2F**).

**Figure 2:**
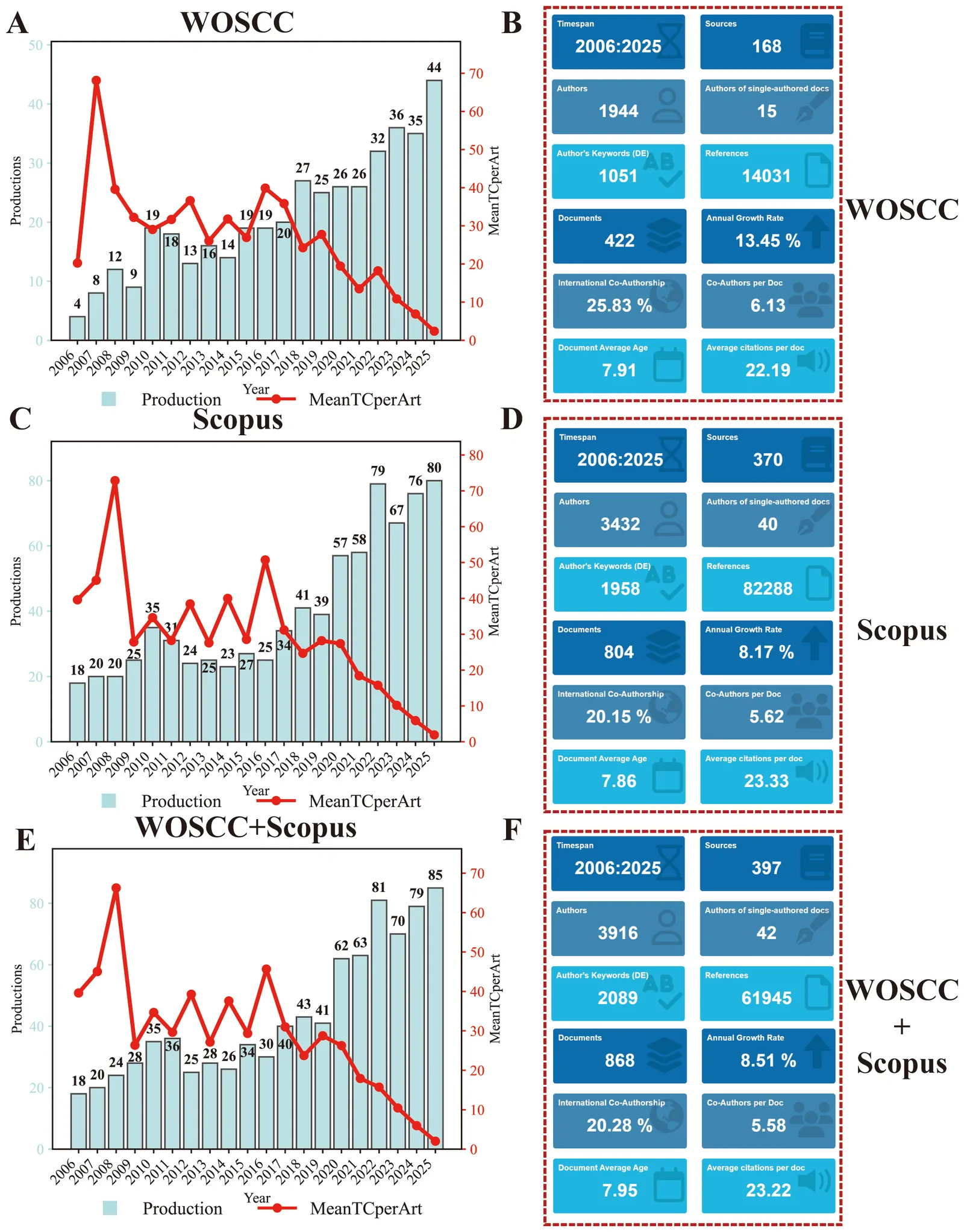
Publication trends and bibliometric overview of the research field. **(A)** Annual publications and citations in the WOSCC database. **(B)** Overall publication and citation profile in WOSCC. **(C)** Annual publications and citations in the SCOPUS database. **(D)** Overall publication and citation profile in SCOPUS. **(E)** Annual publications and citations in the integrated dataset (WOSCC + SCOPUS). **(F)** Overall publication and citation profile in the integrated dataset.

### 3.2 Publication trends and future projections

Among the included publications, original research articles constituted the majority (618/868, 71.20%), whereas review articles accounted for 28.80% (250/868) (**Figure 3A**). To further characterize temporal trends in publication output, ordinary least squares (OLS)-based cubic polynomial regression models were constructed using annual publication data from 2006 to 2025. The coefficients of determination (R^2^) for all three datasets exceeded 0.90, indicating a sustained increase in publication output over the past two decades, with evidence of accelerated growth in recent years (**Figure 3B**). Heatmap analysis further illustrated this pattern, showing a marked increase in publication activity after 2019 and reaching a peak of 85 publications in 2025 (**Figure 3B**).

**Figure 3:**
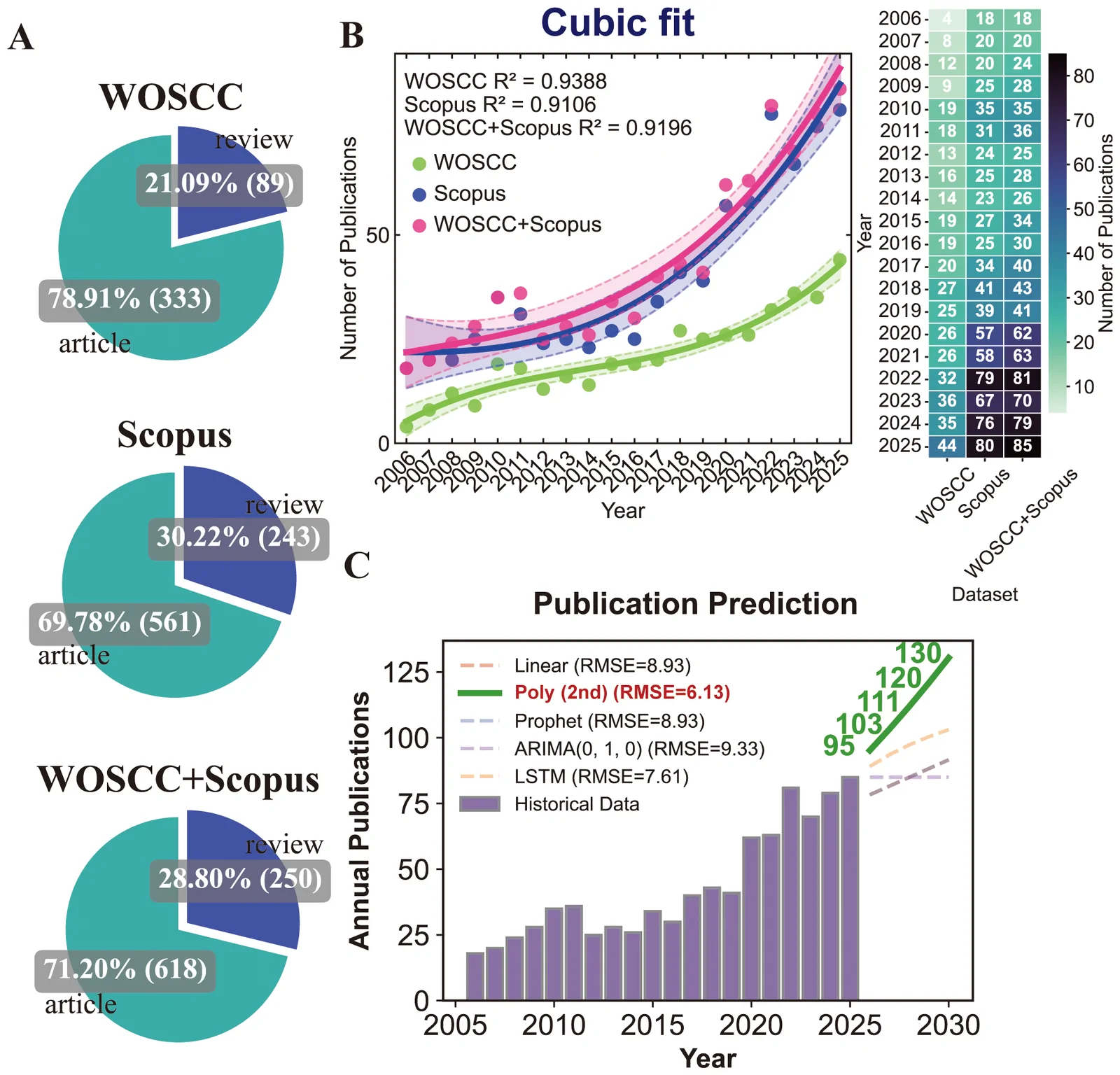
Publication types, trend fitting, and future forecasting. **(A)** Distribution of document types (articles vs. reviews) across WOSCC, Scopus, and the integrated dataset. Articles represent the majority (>70%) in all databases. **(B)** Cubic polynomial regression curves showing the accelerating growth trend of annual publications from 2006 to 2025 for WOSCC, Scopus, and the combined dataset. The high R^2^ values indicate a strong model fit. The adjacent heatmap details the specific publication counts per year. **(C)** Forecasting of future publication output using various models. The second-order polynomial model (Poly 2nd) was identified as the best fit (lowest RMSE), predicting a continued rapid increase in research output, potentially reaching approximately 130 publications annually by 2030.

To predict future research activity, five forecasting models, including Linear, Polynomial (2nd order), Prophet, ARIMA, and Long Short-Term Memory (LSTM), were employed and evaluated using the root mean square error (RMSE) (**Figure 3C**). Among them, the second-order polynomial model achieved the lowest RMSE value (6.13), indicating the best predictive performance, and was therefore selected as the primary forecasting model. Based on this model, the annual number of publications is projected to increase from approximately 95 to 130 over the next five years. Although some variability existed among the different models, all consistently predicted a continued upward trend in publication output.

### 3.3 Core journals and author analysis

The 868 publications were distributed across 397 journals and authored by 3,916 researchers. We further identified the top ten journals with the highest H-index values (**Table 2**). Among them, Otology & Neurotology exhibited the highest H-index (H-index = 16) and published the largest number of articles (n = 37), followed by Acta Oto-Laryngologica (H-index = 13, n = 35) and Frontiers in Neurology (H-index = 13, n = 35) (**Figure 4A**; **Table 2**). These findings indicate that otology, neurotology, and neurology journals constitute the principal publication venues for research on immune-inflammatory mechanisms in MD.

**Table 2:** Top 10 journals ranked by bibliometric indicators.

Author	h_index	g_index	m_index	TC	NP	PY_start
LOPEZ-ESCAMEZ JOSE A.	18	25	1.059	1031	25	2010
SOTO-VARELA ANDRES	14	16	0.875	773	16	2011
ARAN ISMAEL	13	15	0.813	752	15	2011
STRUPP MICHAEL	13	16	0.619	741	16	2006
FREJO LIDIA	11	12	1	538	12	2016
ISHIYAMA AKIRA	11	18	0.524	330	20	2006
ISHIYAMA GAIL	10	17	0.476	300	17	2006
GALLEGO-MARTINEZ ALVARO	9	12	0.9	414	12	2017
BATUECAS-CALETRIO ANGEL	8	8	0.727	460	8	2016
BRANDT THOMAS	8	8	0.381	555	8	2006

**Figure 4:**
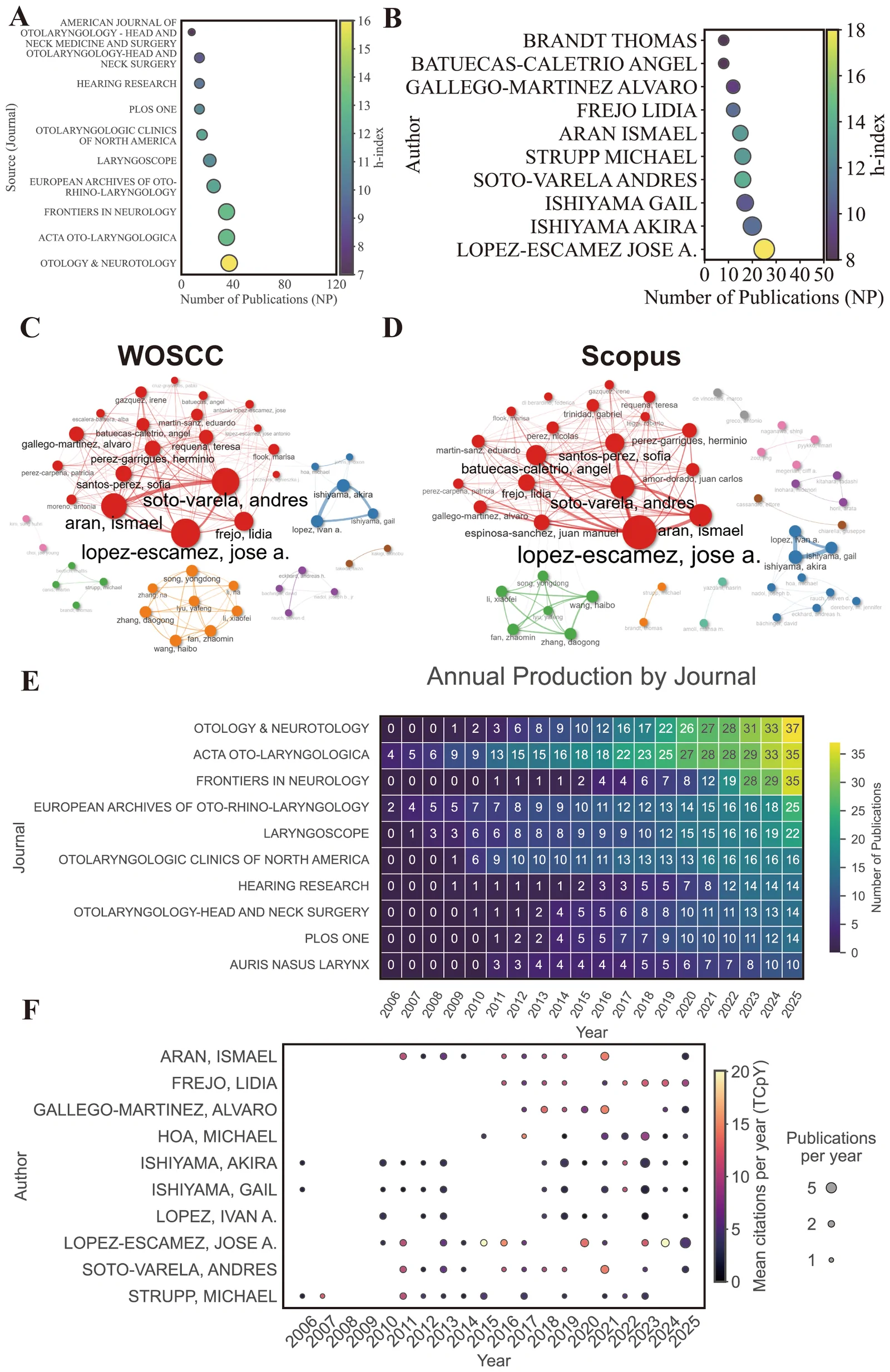
Analysis of journals, authors, and collaboration networks. **(A)** Top 10 most productive journals. Publication count is shown on the x-axis, and bubble size/color indicate H-index. Otology & Neurotology and Acta Oto-Laryngologica are leading journals. **(B)** Top 10 most productive authors. Publication count (x-axis) and H-index (size/color) are displayed. López-Escámez José A. and Soto-Varela Andrés are key contributors. **(C)** Author co-authorship network (WOSCC). Node size reflects publication output, and links indicate collaborations. **(D)** Author co-authorship network (Scopus). Node size reflects publication output, and links indicate collaborations. **(E)** Annual publication trends of the top 10 journals (2006–2025), showing steady growth. **(F)** Annual output and mean citations of top authors, highlighting sustained productivity and impact.

The top ten authors ranked by H-index were identified (**Table 3**). Among all authors, Jose A. Lopez-Escamez had the highest H-index (Arruda et al., 2022) and the largest publication output (n = 25), followed by Andres Soto-Varela (H-index = 14, n = 16), Ismael Aran (H-index = 13, n = 15), and Michael Strupp (H-index = 13, n = 16) (**Table 3**; **Figure 4B**).

**Table 3:** Top 10 authors ranked by bibliometric indicators.

Source	h_index	g_index	m_index	TC	NP	PY_start
OTOLOGY & NEUROTOLOGY	16	25	0.889	672	37	2009
ACTA OTO-LARYNGOLOGICA	13	21	0.619	502	35	2006
FRONTIERS IN NEUROLOGY	13	28	0.813	826	35	2011
EUROPEAN ARCHIVES OF OTO-RHINO-LARYNGOLOGY	12	20	0.571	422	25	2006
OTOLARYNGOLOGIC CLINICS OF NORTH AMERICA	12	16	0.667	435	16	2009
LARYNGOSCOPE	11	22	0.55	1052	22	2007
PLOS ONE	11	14	0.688	495	14	2011
HEARING RESEARCH	10	14	0.556	325	14	2009
OTOLARYNGOLOGY-HEAD AND NECK SURGERY	9	14	0.529	243	14	2010
AMERICAN JOURNAL OF OTOLARYNGOLOGY - HEAD AND NECK MEDICINE AND SURGERY	7	8	0.333	166	8	2006

Author collaboration network analysis further revealed that Jose A. Lopez-Escamez, Andres Soto-Varela, and Ismael Aran occupied central positions within the collaboration networks derived from both databases, forming closely connected research clusters (**Figures 4C, D**). Overall, however, the collaboration network remained relatively fragmented, with most collaborations occurring within specific countries, institutions, or research groups.

The temporal distribution of publications across major journals was analyzed (**Figure 4E**). Otology & Neurotology and Acta Oto-Laryngologica maintained consistently high publication outputs throughout the study period, publishing 37 and 35 articles in 2025, respectively. Frontiers in Neurology exhibited a marked increase in publications after 2018 and reached 35 publications in 2025, becoming one of the major publication platforms in this field. In contrast, Laryngoscope and European Archives of Oto-Rhino-Laryngology showed more gradual increases, with 22 and 25 publications in 2025, respectively. Overall, publication output in most core journals increased steadily after 2010.

The temporal dynamics of scientific productivity and citation impact among leading authors are presented (**Figure 4F**; **Supplementary Table 2**). Jose A. Lopez-Escamez, Ismael Aran, Andres Soto-Varela, and Michael Strupp maintained high publication activity across multiple years and represented the most productive researchers in this field. Among them, Jose A. Lopez-Escamez demonstrated particularly strong academic influence. His publications in 2015 and 2016 accumulated 241 and 162 citations, respectively, corresponding to annual citation rates (TCpY) of 20.08 and 14.73. Notably, his three publications in 2024 achieved a TCpY of 20, indicating sustained citation impact.

Ismael Aran and Andres Soto-Varela exhibited highly similar publication and citation trajectories. Both authors published two articles in 2011, which collectively received 168 citations, and reached another peak in productivity in 2021 with three publications each. In recent years, Alvaro Gallego-Martinez and Lidia Frejo have emerged as active contributors. Gallego-Martinez published two and three articles in 2018 and 2021, respectively, with TCpY values of 12.89 and 14.17, whereas Frejo maintained continuous publication activity since 2016 and achieved TCpY values of approximately 12 during 2022–2024 (**Figure 4F**; **Supplementary Table 2**). In addition, although Akira Ishiyama and Gail Ishiyama published fewer articles annually, they maintained stable research output over an extended period and both achieved 54 citations (TCpY = 10.8) in 2022, reflecting sustained academic influence (**Supplementary Table 2**).

### 3.4 Country analysis

In terms of national contributions, the United States was the leading contributor to research on immune-inflammatory mechanisms in Ménière’s disease, with 189 publications (21.8% of the total). It was followed by China (119, 13.7%), Italy (64, 7.4%), and Japan (61, 7.0%), indicating that these countries constitute the main research contributors in this field (**Figure 5A**; **Table 4**). Regarding international collaboration, among the top ten productive countries, the United States produced the largest number of multi-country publications (MCP = 31), although its MCP ratio was 16.4%. In contrast, Australia, despite a relatively lower total publication output, exhibited the highest MCP ratio (51.4%), suggesting a greater reliance on international collaboration. Spain also showed a relatively high MCP ratio (34.9%, n = 15). These findings indicate notable differences in collaboration patterns across countries.

**Figure 5:**
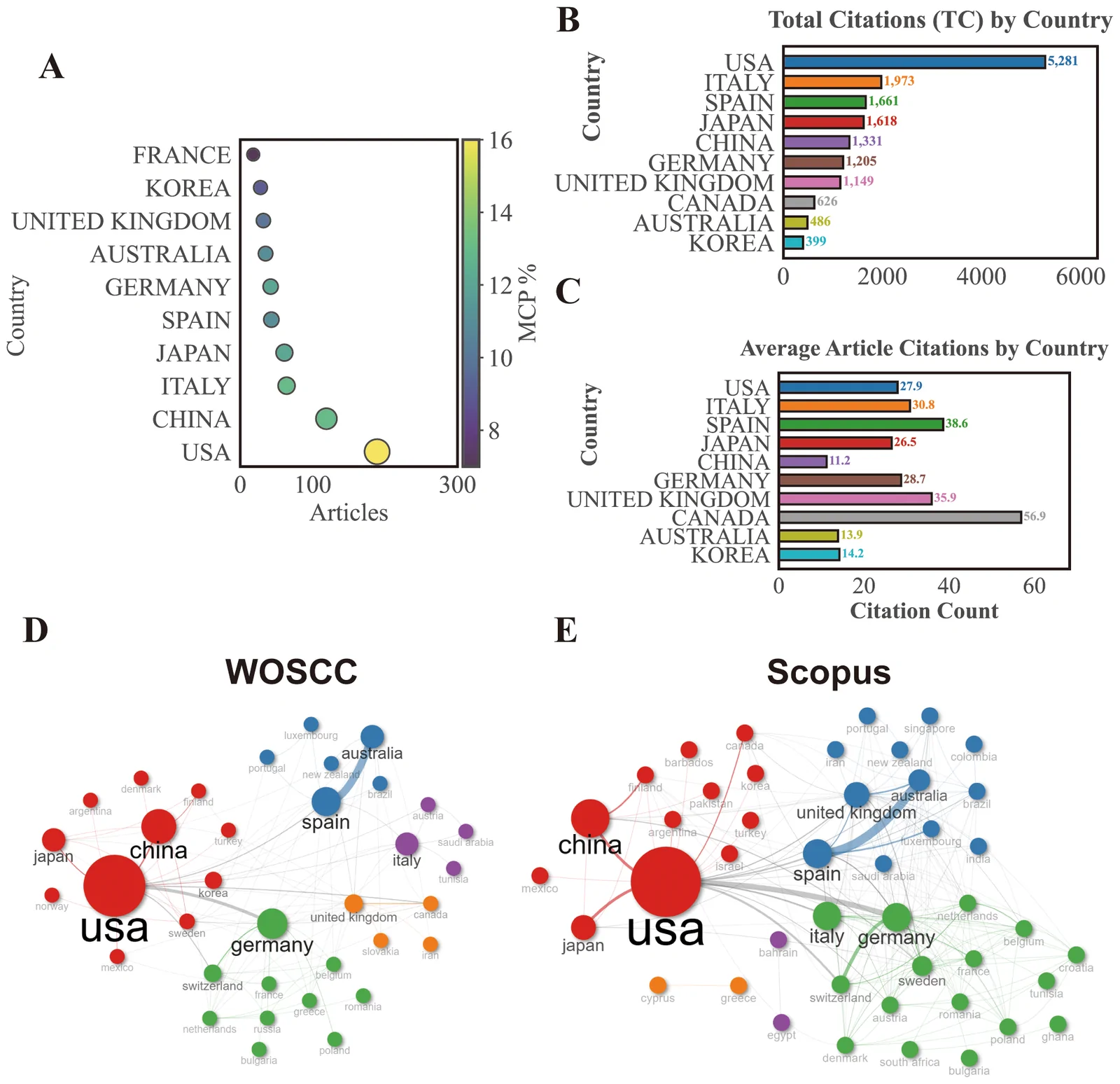
Country-level analysis and collaboration networks. **(A)** Bubble plot of the top 10 productive countries. Publication volume is shown on the x-axis, and MCP% is indicated by color. The USA shows the highest output, while European countries exhibit higher collaboration levels. **(B)** Total citations of the top 10 countries. The USA ranks highest in overall citation impact. **(C)** Average citations per article of the top 10 countries. Canada and the UK show higher citation efficiency. **(D)** Country co-authorship network (WOSCC). Node size reflects publication output, and links indicate collaborations, with the USA, China, and European countries as major hubs. **(E)** Country co-authorship network (Scopus). Node size reflects publication output, and links indicate collaborative relationships, showing similar global cooperation patterns.

**Table 4:** Top 10 countries by publication output and collaboration rate.

Country	Articles	Articles %	SCP	MCP	MCP %
USA	189	21.8	158	31	16.4
CHINA	119	13.7	104	15	12.6
ITALY	64	7.4	50	14	21.9
JAPAN	61	7	55	6	9.8
SPAIN	43	5	28	15	34.9
GERMANY	42	4.8	30	12	28.6
AUSTRALIA	35	4	17	18	51.4
UNITED KINGDOM	32	3.7	23	9	28.1
KOREA	28	3.2	27	1	3.6
FRANCE	18	2.1	13	5	27.8

In terms of citation impact, publications from the United States received the highest total citations (TC = 5,281), followed by Italy (TC = 1,973), Spain (TC = 1,661), and Japan (TC = 1,618) (**Figure 5B**). When normalized by publication output, Canada ranked first in average citations per article (56.9), followed by Spain (38.6) and the United Kingdom (35.9), whereas the United States recorded 27.9 citations per article (**Figure 5C**). These results indicate that while the United States dominates in overall research output and influence, several countries demonstrate higher citation efficiency at the article level.

Country-level collaboration network analysis showed that the United States occupied a central position in both databases and maintained extensive collaborative links with multiple countries (**Figures 5D, E**). China, Germany, Spain, Japan, and Italy also demonstrated relatively high connectivity within the global collaboration network. Overall, international collaboration in this field has developed steadily, although differences in collaboration intensity among countries remain evident.

### 3.5 Institutional analysis

Institutional productivity analysis identified the University of Granada as the most productive institution in this field (n = 58), followed by Harvard University (n = 55), the University of California System (n = 47), and the University of Munich (n = 39) (**Figure 6A**). These institutions represent the principal academic contributors to research on immune-inflammatory mechanisms in Ménière’s disease. Temporal distribution analysis of institutional output showed a progressive increase in research activity from 2019 onwards, particularly for the University of Granada, Harvard University, and the University of California System (**Figure 6B**). After 2020, these institutions maintained sustained growth in publication output, indicating a continued concentration of research activity in recent years.

**Figure 6:**
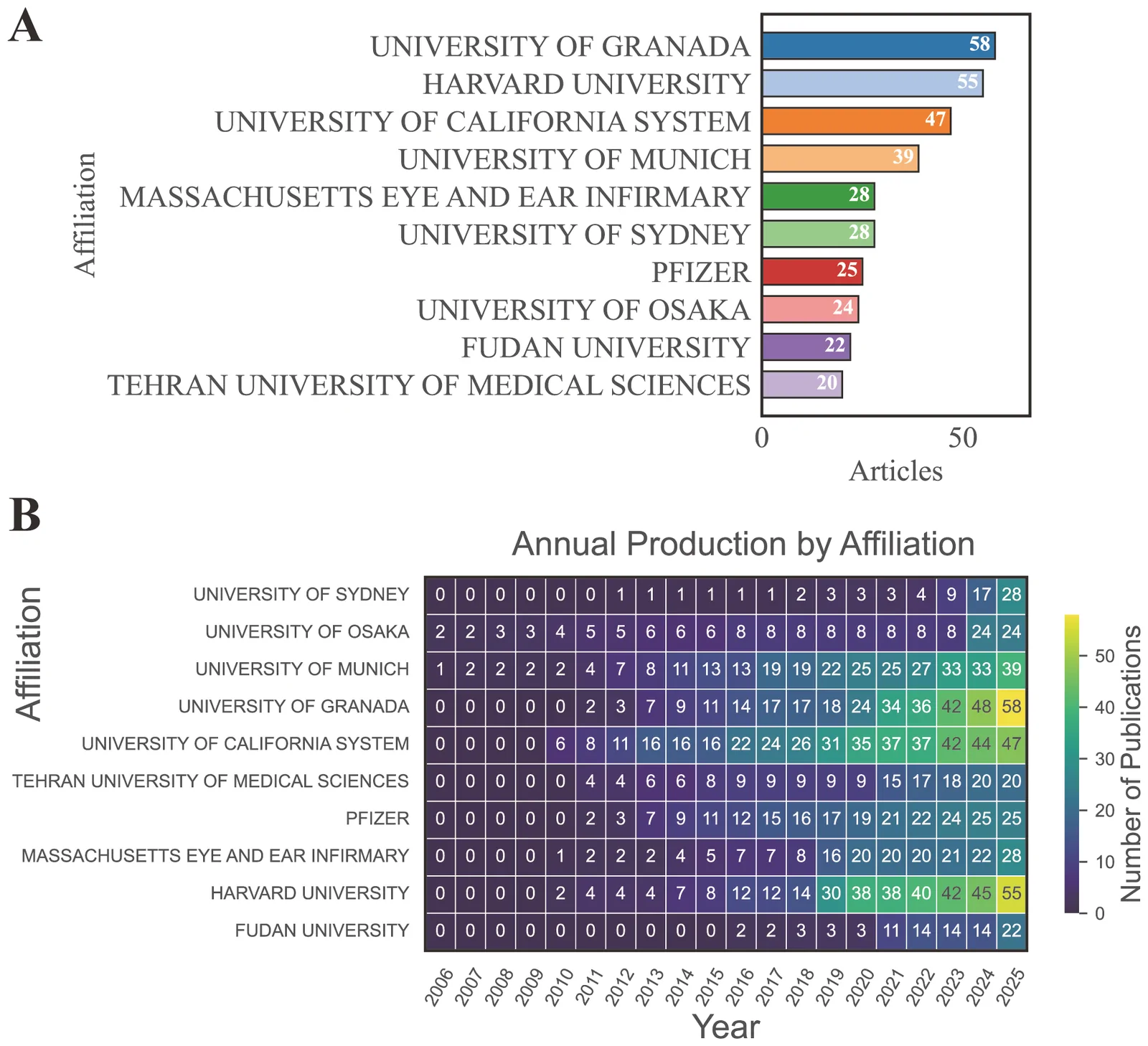
Institutional analysis and temporal productivity. **(A)** Bar chart of the top 10 most productive institutions by publication count. The University of Granada leads with 58 articles, followed by Harvard University (Flook et al., 2023) and the University of California System (Cabrera et al., 2014). **(B)** Heatmap showing annual publication trends for these institutions from 2006 to 2025. Color intensity indicates output volume. University of Granada and Harvard University demonstrate consistent growth, particularly in recent years, while others show more variable activity patterns.

### 3.6 Keyword structure and research hotspot identification

Keyword co-occurrence and thematic evolution analyses were performed to characterize the knowledge structure and research dynamics of immune-inflammatory mechanisms in Ménière’s disease. The keyword clustering network demonstrated a clear modular structure (Modularity Q = 0.4879 > 0.3) and high internal consistency (Weighted mean silhouette S = 0.793 > 0.7), indicating a robust and reliable clustering structure (**Figure 7A**). Eight major clusters were identified, including “#1 cochlea”, “#2 Ménière’s disease”, “#3 noise injury”, “#4 vasculitis”, “#5 vestibular migraine”, “#6 hearing loss”, “#7 urticaria”, and “#8 endolymphatic hydrops” (**Figure 7A**). These clusters primarily encompass inner ear and cochlear pathology, core clinical phenotypes of MD, auditory–vestibular dysfunction, endolymphatic hydrops, and immune-related or differential diagnostic conditions such as vasculitis, urticaria, and vestibular migraine. “Ménière’s disease” (n = 674) was the most frequently occurring keyword, followed by “inner ear” (n = 206), “hearing loss” (n = 173), “vertigo” (n = 159), and “endolymphatic hydrops” (n = 146) (**Figure 7B**), indicating that MD and inner ear dysfunction constitute the central framework of this research field.

**Figure 7:**
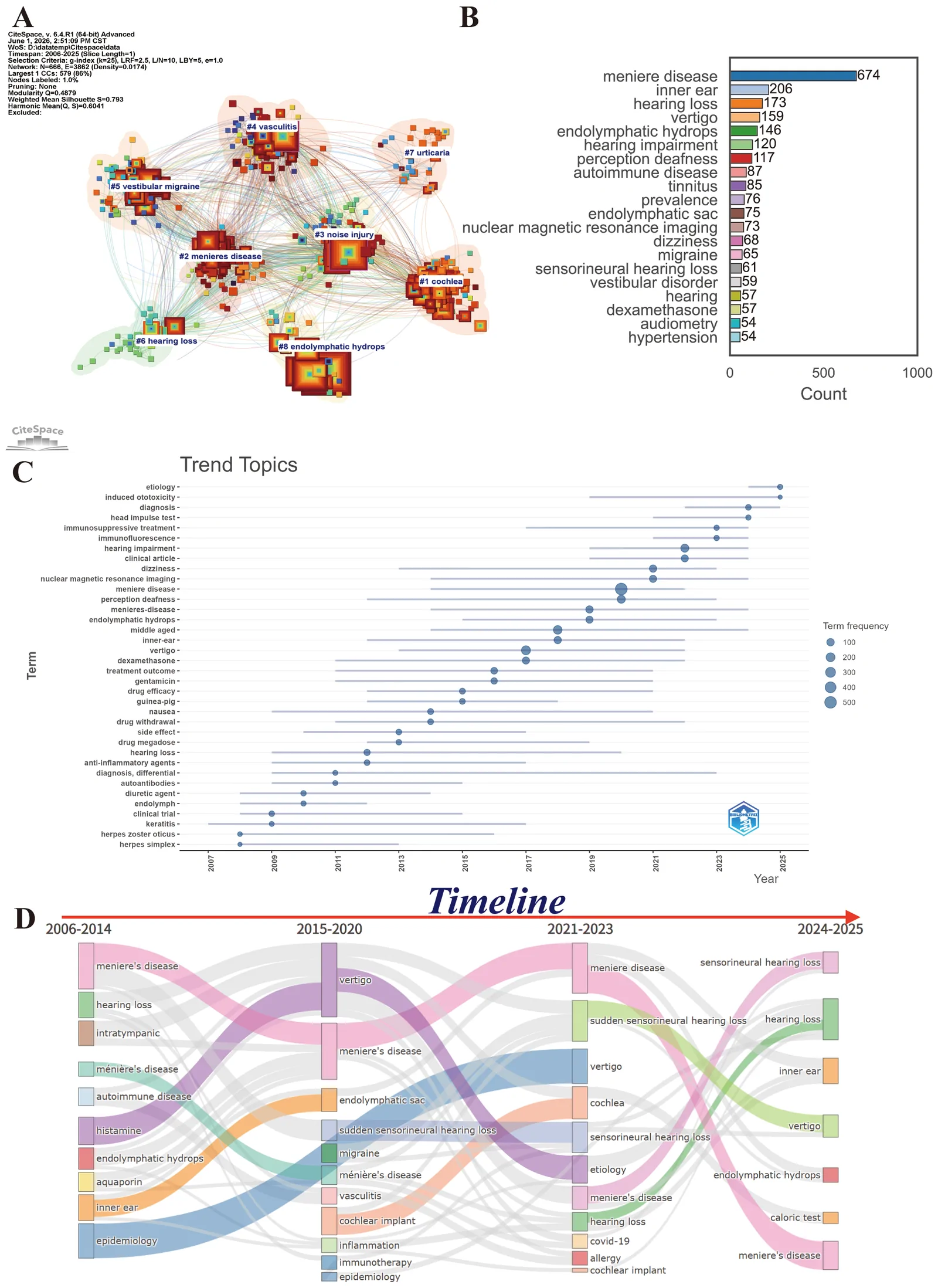
Keyword analysis and thematic evolution. **(A)** Keyword co-occurrence network (k=25) identifying major research clusters such as Meniere’s disease, cochlea, noise injury, and vasculitis. Node size represents frequency, while color indicates the average publication year. **(B)** Top 20 high-frequency keywords. “Meniere disease” is the most prominent term (674 occurrences), followed by “inner ear,” “hearing loss,” and “vertigo.” **(C)** Timeline view of keyword trends from 2006 to 2025. Research focus has shifted from early topics like “etiology” and “diagnosis” to recent hotspots including “dexamethasone,” “immunofluorescence,” and “autoantibodies.” **(D)** Sankey diagram illustrating the evolution of research themes across four time periods (2006–2025). It visualizes how core topics like “Meniere’s disease” and “vertigo” have persisted and branched into emerging areas such as “sudden sensorineural hearing loss” and “cochlear implant.”.

Thematic evolution analysis revealed a stepwise shift in research focus over time (**Figures 7C, D**). From 2006 to 2014, research was mainly concentrated on clinical characterization and local therapeutic approaches, with high-frequency terms including “hearing loss”, “differential diagnosis”, “anti-inflammatory agents”, “intratympanic”, “autoimmune disease”, “endolymphatic hydrops”, “aquaporin”, and “inner ear”. This period was characterized by emphasis on diagnostic classification, auditory phenotypes, endolymphatic hydrops, and intratympanic therapy, alongside emerging attention to autoimmune processes, aquaporin regulation, and inner ear inflammation. Between 2015 and 2020, research emphasis shifted toward treatment evaluation and mechanistic exploration. Frequently occurring terms included “drug efficacy”, “gentamicin”, “dexamethasone”, “treatment outcome”, “vertigo”, and “endolymphatic hydrops”. This phase reflected increased focus on pharmacological interventions, intratympanic therapies, and clinical outcomes. In parallel, the appearance of “vasculitis”, “immunotherapy”, and “inflammation” indicated growing attention to immune-mediated mechanisms and systemic inflammatory involvement in MD. From 2021 to 2023, the field expanded toward multimodal and cross-disease perspectives. Prominent keywords included “nuclear magnetic resonance imaging”, “immunofluorescence”, “immunosuppressive treatment”, “cochlea”, “sensorineural hearing loss”, “sudden sensorineural hearing loss”, “etiology”, “allergy”, and “COVID-19”, reflecting increased integration of imaging-based assessment, immunological validation, and systemic or infectious factors in MD research. In 2024–2025, research attention further concentrated on etiological integration, diagnostic refinement, and functional assessment. Keywords such as “etiology”, “diagnosis”, “head impulse test”, and “caloric test” highlight increasing interest in objective diagnostic stratification. Core terms including “inner ear”, “endolymphatic hydrops”, “hearing loss”, and “vertigo” remained consistently prominent, indicating the sustained centrality of inner ear dysfunction and vestibular–auditory impairment in this field.

### 3.7 Citation analysis

To delineate the knowledge base and key scholarly contributors in the field of immunoinflammatory mechanisms in Ménière’s disease (MD), a co-citation network of authors was constructed using VOSviewer (**Figure 8A**). The co-citation analysis identified Lopez-Escamez, Jose A., Soto-Varela, Andres, and Aran, Ismael as central nodes within the network, indicating that their work has been extensively co-cited and has exerted substantial influence on subsequent studies in the field.

**Figure 8:**
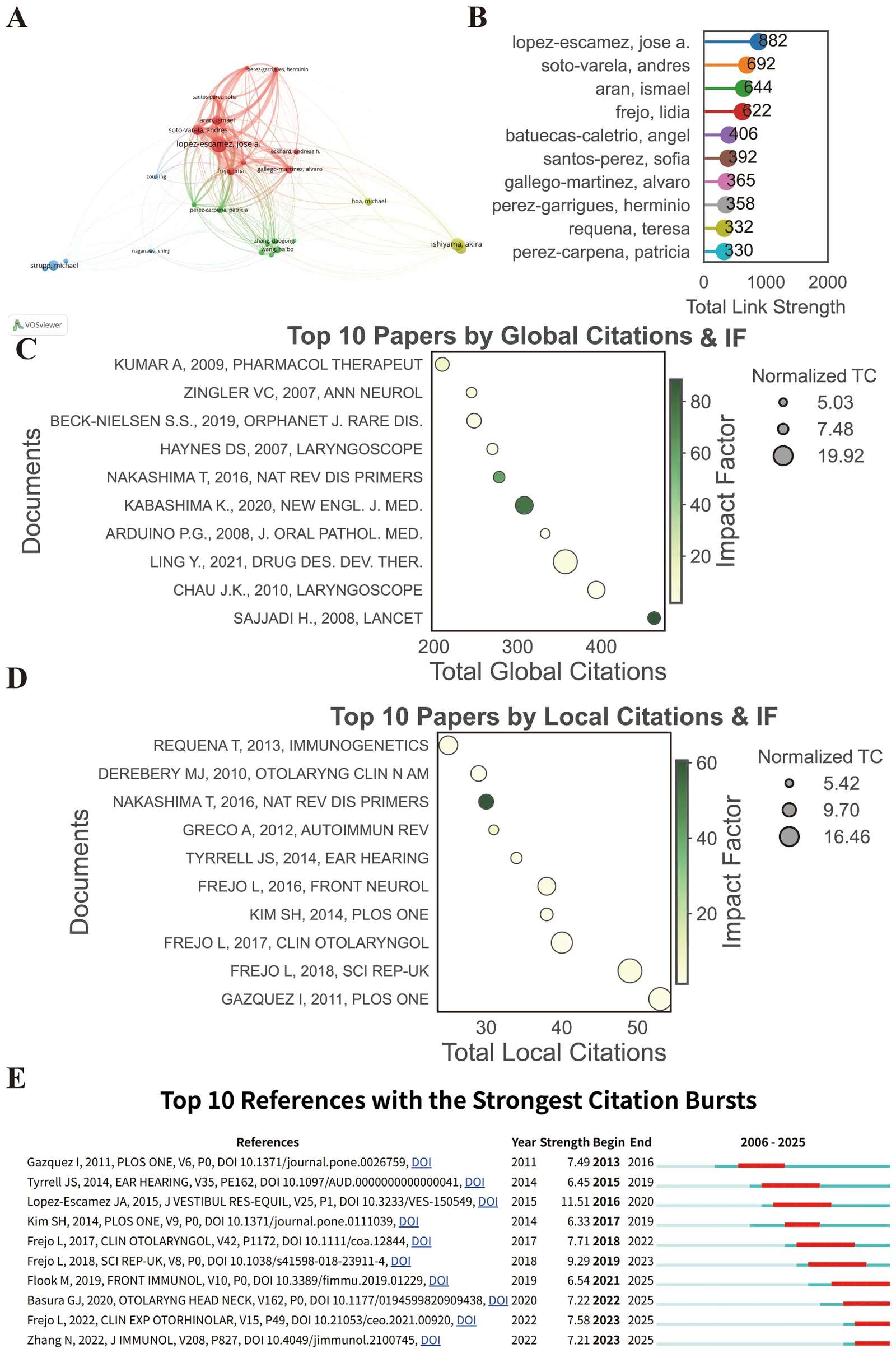
Citation analysis and research impact. **(A)** Author co-citation network visualization generated by VOSviewer, revealing distinct clusters of influential researchers and collaborative groups within the field. Node size indicates citation frequency. **(B)** Top 10 authors ranked by total link strength in the co-citation network. Lopez-Escamez JA (882) and Soto-Varela A (692) emerge as the most central figures in the literature. **(C)** Bubble plot of the top 10 most globally cited publications versus Journal Impact Factor (IF). Bubble size represents normalized total citations. Key high-impact papers include works by Kumar A (2009) and Zingler VC (2007). **(D)** Bubble plot of the top 10 publications ranked by local citations and IF. This highlights foundational papers within the specific dataset, such as Requena T (2013) and Nakashima T (2016), which are critical to the core research community. **(E)** Top 10 references with the strongest citation bursts from 2006 to 2025. The red segments indicate periods of intense citation activity, highlighting emerging trends and pivotal studies like Lopez-Escamez JA (2015) and recent works by Frejo L and Zhang N.

Quantitatively, Lopez-Escamez, Jose A. demonstrated the highest total link strength (882), followed by Soto-Varela, Andres (692), Aran, Ismael (644), and Frejo, Lidia (622) (**Figure 8B**). These highly co-cited authors have long focused on the clinical phenotyping, genetic susceptibility, immunoinflammatory mechanisms, and inner ear pathology of MD, collectively forming a foundational intellectual framework for the field.

In addition, the top 10 most frequently cited publications in the MD immunoinflammation research domain were identified using the R-Bibliometrix package (**Table 5**; **Figure 8C**). The most cited article was published by Sajjadi H. et al. in The Lancet (2008), with a total of 463 citations and a normalized citation score of 6.99. This study comprehensively summarized the clinical features, diagnostic criteria, pathophysiological mechanisms, and therapeutic strategies of MD, and has served as a cornerstone reference in the field (Sajjadi and Paparella, 2008). The second most cited work was published by Chau J.K. et al. in The Laryngoscope (2010), with 394 citations and a normalized citation score of 11.37 (Chau et al., 2010) (Ling et al., 2021). published a study in Drug Design, Development and Therapy that accumulated 357 citations (normalized score: 19.92) (Ling et al., 2021) (Arduino and Porter, 2008), published in the Journal of Oral Pathology & Medicine, received 333 citations (normalized score: 5.03) (Arduino and Porter, 2008). Collectively, these highly cited works primarily address disease characterization, etiological hypotheses, inner ear pathology, clinical complications, and therapeutic strategies, thereby establishing the foundational knowledge structure of the field.

**Table 5:** Top 10 globally most cited publications.

Paper	DOI	Total citations	TC per Year	Normalized TC	IF
SAJJADI H., 2008, LANCET	10.1016/S0140-6736(08)61161-7	463	24.37	6.99	88.5
CHAU J.K., 2010, LARYNGOSCOPE	10.1002/lary.20873	394	23.18	11.37	1.9
LING Y., 2021, DRUG DES. DEV. THER.	10.2147/DDDT.S329547	357	59.50	19.92	5.1
ARDUINO P.G., 2008, J. ORAL PATHOL. MED.	10.1111/j.1600-0714.2007.00586.x	333	17.53	5.03	2.3
KABASHIMA K., 2020, NEW ENGL. J. MED.	10.1056/NEJMoa1917006	308	44.00	11.73	78.5
NAKASHIMA T, 2016, NAT REV DIS PRIMERS	10.1038/nrdp.2016.28	278	25.27	6.09	60.6
HAYNES DS, 2007, LARYNGOSCOPE	10.1097/01.mlg.0000245058.11866.15	270	13.50	5.99	1.9
BECK-NIELSEN S.S., 2019, ORPHANET J. RARE DIS.	10.1186/s13023-019-1014-8	248	31.00	8.62	3.5
ZINGLER VC, 2007, ANN NEUROL	10.1002/ana.21105	245	12.25	5.44	7.7
KUMAR A, 2009, PHARMACOL THERAPEUT	10.1016/j.pharmthera.2009.07.003	210	11.67	7.97	12.5

To further characterize the internal knowledge structure of the dataset, the top 10 publications ranked by local citations are shown in a bubble chart (**Figure 8D**; **Table 6**).Local citations refer to the number of citations received within the set of MD immune-inflammatory literature included in this study. Compared with global citations, which reflect overall impact across the scientific literature, local citations better capture the foundational and field-specific contributions within this research domain (Gazquez et al., 2011). showed a high number of local citations. This multicenter cohort study reported an increased prevalence of systemic autoimmune diseases, including rheumatoid arthritis, systemic lupus erythematosus, and ankylosing spondylitis, in patients with MD, supporting an autoimmune-related hypothesis of MD (Gazquez et al., 2011; Frejo et al., 2018). also exhibited a high local citation frequency and a large bubble size (representing normalized total citations, Normalized TC). This study demonstrated elevated baseline levels of IL-1β, IL-6, and TNF-α in peripheral blood mononuclear cells from MD patients, as well as enhanced TNF-α release and distinct gene expression responses following stimulation with Aspergillus and Penicillium, suggesting a pro-inflammatory immune phenotype in a subset of patients (Frejo et al., 2018). These studies provide complementary evidence from epidemiological and immunological perspectives supporting the immune-inflammatory features of MD and represent frequently cited works within the analyzed literature set.

**Table 6:** Top 10 locally most cited publications.

Paper	DOI	Local citations	Normalized local citations
GAZQUEZ I, 2011, PLOS ONE	10.1371/journal.pone.0026759	53	15.26
FREJO L, 2018, SCI REP-UK	10.1038/s41598-018-23911-4	49	16.46
FREJO L, 2017, CLIN OTOLARYNGOL	10.1111/coa.12844	40	13.56
KIM SH, 2014, PLOS ONE	10.1371/journal.pone.0111039	38	6.91
FREJO L, 2016, FRONT NEUROL	10.3389/fneur.2016.00182	38	10.56
TYRRELL JS, 2014, EAR HEARING	10.1097/AUD.0000000000000041	34	6.18
GRECO A, 2012, AUTOIMMUN REV	10.1016/j.autrev.2012.01.004	31	5.42
NAKASHIMA T, 2016, NAT REV DIS PRIMERS	10.1038/nrdp.2016.28	30	8.33
DEREBERY MJ, 2010, OTOLARYNG CLIN N AM	10.1016/j.otc.2010.05.004	29	8.83
REQUENA T, 2013, IMMUNOGENETICS	10.1007/s00251-013-0683-z	25	11.29

To identify pivotal turning points and emerging research fronts, CiteSpace was employed to conduct citation burst analysis (**Figure 8E**). Citation bursts refer to a rapid and substantial increase in citations to a given publication within a specific period, often indicating heightened scholarly attention and signaling shifts in research focus or emerging frontiers (Li et al., 2022). The analysis revealed that the strongest citation burst was associated with (Lopez-Escamez et al., 2015), published in the Journal of Vestibular Research, with a burst strength of 11.51 and a marked increase in citations beginning in 2016. This study, developed by the Bárány Society Classification Committee in collaboration with multiple international expert organizations, established standardized diagnostic criteria for MD, classifying the disease into “definite” and “probable” forms (Lopez-Escamez et al., 2015). This consensus framework has provided a unified foundation for clinical diagnosis, epidemiological research, and mechanistic investigations, and remains one of the most influential contributions in the field. The second strongest citation burst was observed for (Frejo et al., 2018), published in Scientific Reports (burst strength: 9.29). This study reported significantly elevated levels of inflammatory mediators, including IL-1β, IL-1RA, IL-6, and TNF-α, in the peripheral blood of MD patients compared with healthy controls (Frejo et al., 2018), thereby providing robust evidence supporting an immunoinflammatory contribution to MD and facilitating a conceptual shift from a purely hydrops-centric model toward immune-mediated mechanisms. More recent burst publications have predominantly focused on immunoregulatory pathways. A 2022 review by Frejo L. et al. in Clinical and Experimental Otorhinolaryngology (burst strength: 7.58) systematically summarized inflammatory cytokine networks and potential immunotherapeutic targets in MD, highlighting a subset of patients characterized by chronic low-grade inflammation driven by IL-1β and TNF-α dysregulation and proposing candidate agents for immunomodulatory intervention (Frejo and Lopez-Escamez, 2022). Similarly (Zhang et al., 2022b), published in the Journal of Immunology (burst strength: 7.21), further elucidated a potential role of IgE-mediated immune responses in MD pathogenesis, demonstrating that CD23-dependent IgE transcytosis may contribute to the initiation of local inner ear inflammatory processes, thereby implicating allergic immune pathways as a potential mechanistic contributor to disease development (Zhang et al., 2022b).

We further conducted a dual-map overlay analysis using CiteSpace (**Supplementary Figure 1**). The main citation pathways reflect knowledge inheritance and transfer across disciplines. The most prominent pathway shows that journals in “molecular biology, immunology” on the left predominantly cite journals in “molecular biology, genetics” on the right, indicating that molecular and genetic research provides a key theoretical and mechanistic foundation for immunological studies. In addition, citation flows from “dentistry, dermatology, surgery” toward “molecular biology, genetics” suggest that clinically oriented research increasingly extends into basic molecular mechanisms, reflecting a trend in which clinical observations drive mechanistic investigations. Notably, clear interdisciplinary links are observed between “molecular biology, immunology” and “dentistry, dermatology, surgery,” indicating continuous bidirectional knowledge exchange between clinical medicine and basic life sciences. Overall, the field is characterized by a multidisciplinary structure centered on medicine and life sciences, with strong integration between clinical practice and basic research.

## 4 Discussion

### 4.1 Overall research landscape

This study systematically evaluated the literature on immunoinflammatory mechanisms in Ménière’s disease (MD) published between 2006 and 2025 using the Web of Science Core Collection and Scopus databases. Overall, the publication trajectory demonstrates a sustained and progressively accelerating expansion, indicating that the immunoinflammatory paradigm has evolved from an exploratory hypothesis into an increasingly central framework for understanding the pathophysiology of MD. The included corpus is dominated by original research articles, with a substantial proportion of review papers, suggesting that the field has established a relatively solid empirical foundation while simultaneously entering a phase of conceptual integration and theoretical consolidation.

The continuous increase in annual publication output, with a compound annual growth rate (CAGR) of 8.51%, further indicates that research on immunoinflammatory mechanisms in MD is entering a phase of accelerated development. Notably, a marked surge in publications after 2017, particularly between 2019 and 2022, suggests a rapid expansion of academic interest. This growth may be attributed to an improved understanding of disease heterogeneity, advances in inner ear imaging and molecular immunology, and the broader incorporation of immunology-oriented approaches within otology and neurotology. In addition, the COVID-19 pandemic may have acted as an external driver contributing to this increase in research activity (Lovato et al., 2021; Berlot et al., 2023).

From a journal perspective, publications are primarily concentrated in otology, neurotology, and neurology-oriented journals, with Otology & Neurotology, Acta Oto-Laryngologica, and Frontiers in Neurology serving as the major dissemination platforms. These patterns indicate that the field remains largely anchored within otolaryngology and vestibular medicine, while progressively integrating with neuroscience, immunology, and translational medicine. Although a subset of studies has appeared in general medical journals, the overall distribution remains predominantly specialized, suggesting substantial potential for further interdisciplinary expansion and clinical translation. Analysis of authorship and research groups reveals several relatively stable core teams within the field. Researchers such as Lopez-Escamez, Jose A., Soto-Varela, Andres, Aran, Ismael, and Strupp, Michael occupy central positions in both productivity and collaboration networks, with their work primarily focused on clinical phenotyping, genetic susceptibility, and immunoinflammatory mechanisms in MD.

At the national level, the United States leads in both publication output and total citation impact, reflecting its dominant role in shaping the intellectual landscape of the field. China, Italy, Japan, and Spain also contribute substantially, forming an important multinational research base. At the institutional level, the University of Granada, Harvard University, the University of California system, and the University of Munich represent key hubs with high connectivity and research activity within the collaboration network. Overall, while collaborative ties between research groups are increasingly evident, the network remains largely structured around country- or institution-centric clusters, with relatively limited cross-regional and cross-group integration.

### 4.2 Evolution of research hotspots and emerging frontiers

#### 4.2.1 From endolymphatic hydrops to immunoinflammation

Endolymphatic hydrops has long been regarded as the most characteristic histopathological feature of Ménière’s disease (MD) and has been closely linked to its cardinal symptoms, including vertigo, fluctuating sensorineural hearing loss, tinnitus, and aural fullness (Ishiyama et al., 2015; Nakashima et al., 2016). However, temporal bone pathology and clinical observations have revealed a notable dissociation between hydrops and clinical manifestations: some individuals with endolymphatic hydrops remain asymptomatic, while substantial heterogeneity exists among patients with respect to attack frequency, hearing progression, bilateral involvement, and therapeutic response (Foster and Breeze, 2013; Zhang et al., 2022; Chen et al., 2025). These findings suggest that endolymphatic hydrops is more plausibly a downstream structural outcome of multiple upstream pathological processes rather than a singular primary driver capable of fully accounting for disease heterogeneity (Rizk et al., 2022).

The results of keyword co-occurrence and thematic evolution analyses in the present study are broadly consistent with this interpretation. While endolymphatic hydrops and related structural alterations of the inner ear remain a foundational focus of early research, recent studies have increasingly shifted toward immunoinflammatory mechanisms, etiological integration, and patient stratification. Accordingly, growing attention has been directed to the links between upstream immune-inflammatory processes and disruption of inner ear homeostasis, with efforts to construct integrative explanatory frameworks across different pathological levels. This conceptual shift has, to some extent, redefined the position of endolymphatic hydrops within the pathogenic cascade of MD and supports the view of MD as a clinically and biologically heterogeneous spectrum of immunoinflammatory disorders.

#### 4.2.2 The IL-1β/TNF-α inflammatory axis

In recent years, increasing attention has been paid to cytokine-mediated inflammatory networks in the immunopathogenesis of MD. Frejo et al. reported elevated levels of IL-1β, IL-1RA, IL-6, and TNF-α in peripheral blood mononuclear cells from a subset of MD patients, suggesting that MD may encompass an immunologically distinct subtype characterized by chronic low-grade inflammation or autoinflammatory features (Frejo and Lopez-Escamez, 2022; Frejo et al., 2025).

As key mediators of innate immune responses, IL-1β and TNF-α may contribute to inner ear dysfunction through amplification of inflammatory signaling, recruitment of immune cells, and increased vascular permeability. These processes may disrupt the blood–labyrinth barrier and impair inner ear fluid homeostasis, thereby contributing to endolymphatic dysregulation and vestibular–cochlear dysfunction (Zou et al., 2022; Sekulic et al., 2023). In addition, IL-6, IFN-γ, and associated chemokines may act synergistically to sustain a persistent pro-inflammatory microenvironment within the inner ear (Huang et al., 2022).

From a translational perspective, the IL-1β/TNF-α axis provides a potential framework for immunological stratification in MD. The therapeutic effects of corticosteroids observed in a subset of patients may, at least in part, be mediated through suppression of multiple pro-inflammatory cytokines; however, the marked inter-individual variability in treatment response suggests underlying immunoinflammatory heterogeneity (Missner et al., 2022; Li and She, 2024). At present, evidence supporting cytokine-targeted therapies in MD remains limited, and their efficacy as well as target patient populations require further validation through prospective and stratified clinical studies (Zhang et al., 2022a). Overall, investigation of the IL-1β/TNF-α signaling axis has advanced the understanding of immunoinflammatory mechanisms in MD and highlights potential directions for future studies of disease subtyping and therapeutic target validation.

#### 4.2.3 NF-κB/TLR signaling pathway

The NF-κB/TLR signaling pathway is increasingly recognized as a key component linking innate immune activation to the immunoinflammatory pathogenesis of Ménière’s disease (MD). Toll-like receptors (TLRs), as pattern recognition receptors, respond to infectious stimuli, tissue injury, and endogenous danger-associated signals, and subsequently activate NF-κB through downstream signaling cascades, thereby inducing the expression of IL-1β, IL-6, TNF-α, and a broad spectrum of chemokines (Lopez-Escamez et al., 2018; Salauddin et al., 2025). Compared with the IL-1β/TNF-α inflammatory axis, this pathway more explicitly reflects upstream regulatory control of pro-inflammatory cytokine production (**Figure 9**).

**Figure 9:**
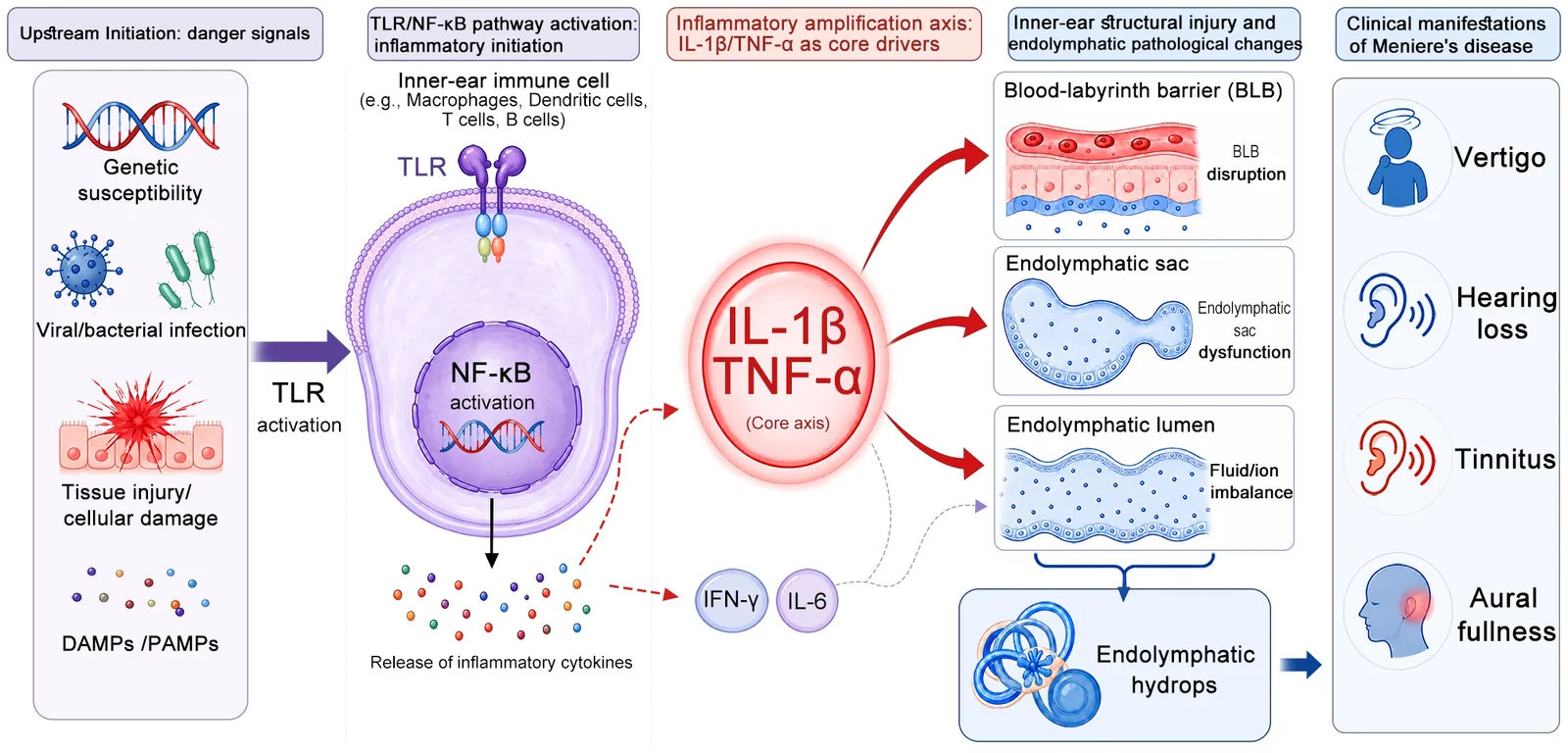
Proposed immunoinflammatory mechanism in Ménière’s disease involving TLR/NF-κB signaling and IL-1β/TNF-α amplification. Genetic susceptibility, endogenous danger signals, and systemic inflammatory background may activate the TLR/NF-κB pathway in the inner ear immune microenvironment. NF-κB activation promotes the release of IL-1β, TNF-α, IL-6, IFN-γ, and chemokines, forming a self-amplifying inflammatory loop. This process may increase blood–labyrinth barrier permeability, impair immune and fluid regulation of the endolymphatic sac, disrupt ionic and fluid homeostasis, and ultimately lead to endolymphatic hydrops, resulting in episodic vertigo, fluctuating hearing loss, tinnitus, and aural fullness.

Genetic evidence further supports its potential involvement in disease susceptibility and progression. Variants in TLR10 and NFKB1 have been associated with bilateral disease involvement, clinical progression, and hearing outcomes in MD (Requena et al., 2013; Cabrera et al., 2014). In addition, Frejo et al. reported that the bilateral MD risk locus rs4947296 may enhance NF-κB-mediated inflammatory activity through modulation of the TWEAK/Fn14 signaling axis, thereby linking genetic susceptibility with inflammatory regulation (Frejo et al., 2017). Mechanistically, NF-κB/TLR signaling may contribute to MD pathophysiology by lowering the activation threshold of innate immune responses, promoting cytokine release, disrupting the blood–labyrinth barrier, and impairing inner ear fluid homeostasis, ultimately contributing to endolymphatic dysregulation and vestibular–cochlear dysfunction (Kämpfe Nordström et al., 2018; Zhang et al., 2024). Variability in pathway activation may also partially underlie heterogeneity in bilateral involvement, disease progression, and treatment response among patients.

#### 4.2.4 IgE/CD23 pathway

The role of allergic mechanisms in MD has long been suggested; however, earlier evidence was largely limited to epidemiological associations, increased prevalence of allergic history, and empirical therapeutic observations, with limited mechanistic resolution (Lopez-Escamez et al., 2023; Zeng et al., 2024). In recent years, investigation of the IgE/CD23 pathway has provided a more refined molecular and cellular framework for this hypothesis.

Zhang et al. reported elevated serum levels of IgE and type 2 inflammatory cytokines, including IL-4, IL-5, IL-10, and IL-13, in MD patients, with IgE concentrations showing correlations with the degree of endolymphatic hydrops and hearing classification (Zhang et al., 2022b). In parallel, IgE deposition has been observed in inner ear-related structures, and expression of the low-affinity IgE receptor CD23 has been detected in cochlear hair cells, suggesting a potential role in IgE trafficking within the inner ear microenvironment (Ma et al., 2021). Mechanistically, the IgE/CD23 axis may serve as a functional bridge between allergic immune responses and local inflammatory processes within the inner ear (Palaniyandi et al., 2015).

Type 2 cytokines such as IL-4 may modulate CD23 expression and, together with IgE-mediated transport processes, contribute to local immune activation and inflammatory mediator release, thereby affecting blood–labyrinth barrier integrity and inner ear fluid homeostasis (Flook et al., 2023). In contrast to the innate immune-dominant IL-1β/TNF-α axis, the IgE/CD23 pathway more specifically reflects the potential involvement of allergy-associated immune responses in a subset of MD patients, further supporting the existence of immunological heterogeneity within the disease spectrum.

### 4.3 Future perspectives and recommendations

The present findings indicate that immunoinflammatory mechanisms have become an increasingly prominent direction in Ménière’s disease (MD) research. Compared with earlier studies that primarily focused on endolymphatic hydrops and clinical phenotypes, recent work has increasingly highlighted the potential roles of inflammatory mediators, innate immune signaling, allergy-associated responses, and patient heterogeneity in disease initiation and progression. This shift suggests that MD may not be adequately explained by a single structural lesion alone, but instead should be conceptualized within the context of distinct immunoinflammatory endotypes.

From a methodological perspective, patient stratification is likely to represent a key avenue for future research. Accumulating evidence indicates substantial inter-individual variability in attack frequency, hearing deterioration, bilateral involvement, allergic history, and autoimmune background. These differences may reflect underlying immunoinflammatory heterogeneity. Accordingly, integrating cytokine profiles, IgE-related biomarkers, genetic susceptibility, imaging features, and vestibular–auditory functional assessments may enable a more refined characterization of disease subtypes.

Recent epigenomic and multi-omic findings also have important implications for future patient stratification. DNA methylation-based clustering and single-cell transcriptomic/ATAC-seq analyses suggest that molecular subgroups of MD may be defined by distinct combinations of inflammatory activity, immune-cell composition, transcriptional programs, and chromatin states (Flook et al., 2024; Patil et al., 2025). These findings provide a biological basis for moving beyond phenotype-based classification toward molecularly informed endotyping. Future studies should therefore consider integrating cytokine profiles, DNA methylation, chromatin accessibility, transcriptomics, immune-cell composition, genetic susceptibility, and clinical phenotypes within the same patients. Such longitudinal and multimodal designs will be particularly important for determining whether molecular endotypes remain stable over time, correspond to specific clinical trajectories, or predict therapeutic responses.

Recent mechanistic studies further suggest that these immune states may have functional consequences within the inner ear. For example, IL-1β has been shown to promote glutaminase expression and glutamate accumulation, providing a potential link between inflammatory signaling and synaptic dysfunction in MD (Zhang et al., 2024). In parallel, recent experimental evidence implicates cGAS-STING activation in vestibular macrophages as a mediator linking oxidative stress and sensory epithelial injury to innate immune activation (Liu et al., 2026). Although these studies do not directly establish the molecular clusters identified by epigenomic studies, they support the broader concept that distinct immune regulatory states may converge on different pathological mechanisms in the inner ear.

From a translational standpoint, the relationship between immunoinflammatory markers and clinical outcomes remains incompletely defined. Although IL-1β, TNF-α, IL-6, IgE, and NF-κB/TLR-associated pathways have been implicated in MD pathophysiology, their robustness in predicting disease activity, prognosis, and therapeutic response requires further validation. Current treatment strategies remain largely empirical, and variable responses to corticosteroids, immunomodulatory agents, and anti-allergic therapies further underscore the need for stratified therapeutic approaches aligned with underlying biological heterogeneity.

Finally, given the clinical and immunological complexity of MD, limitations inherent to single-center and small-sample studies remain a major challenge for result reproducibility. Future investigations should prioritize multicenter collaboration with standardized diagnostic criteria, harmonized assay platforms, and unified follow-up endpoints, which would substantially improve comparability across studies and enhance the robustness of evidence generation.

## 5 Limitations and study constraints

This study has several limitations that warrant consideration. First, only English-language publications were included. While this improves consistency and comparability across datasets, it may have led to the exclusion of relevant studies published in other languages, such as Chinese, German, or Japanese. Nevertheless, non-English literature generally has more limited visibility and citation dissemination within the global research landscape; therefore, the present findings mainly reflect the dominant international body of literature. Second, although a comprehensive search strategy was applied using multiple terms related to immunity, inflammation, autoimmunity, allergy, cytokines, and immunotherapy, and data were integrated from both the WoSCC and Scopus, incomplete retrieval cannot be fully ruled out. Differences in terminology and database indexing practices may still result in the omission of relevant records. Third, inherent limitations of bibliometric indicators should be acknowledged. Citation-based metrics are influenced by time-dependent effects, whereby earlier publications tend to accumulate more citations, while more recent high-quality studies may not yet have fully accrued their academic impact. Finally, as a bibliometric and visualization-based study, this work primarily maps the knowledge structure, research hotspots, and thematic evolution of MD immunoinflammatory research. It does not provide experimental validation and cannot replace mechanistic studies or clinical investigations. Despite these limitations, the integration of multiple databases and systematic analytical approaches provides a comprehensive overview of the field, offering a useful reference for future mechanistic studies, patient stratification, and translational research in Ménière’s disease.

## 6 Conclusion

This study systematically maps the global research landscape and thematic evolution of immune-inflammatory research in Ménière’s disease from 2006 to 2025 using a multi-database bibliometric and visualization approach. The field has progressively shifted from classical clinical phenotypes and endolymphatic hydrops toward immune dysregulation, inflammatory cytokine networks, innate immune signaling, allergy-associated mechanisms, and patient stratification. These research trends highlight immunoinflammatory heterogeneity and molecular endotyping as emerging hypotheses that warrant further biological and clinical investigation. The identified molecular and immunological pathways may help prioritize future studies of disease mechanisms, biomarker validation, molecular stratification, and targeted therapeutic evaluation. However, the present bibliometric analysis does not establish molecular endotypes, validate biomarkers, or demonstrate the efficacy of precision immunomodulatory treatments; these questions require independent mechanistic studies and prospective, appropriately stratified clinical investigations.

## Data Availability

The original contributions presented in the study are included in the article/**Supplementary Material**, further inquiries can be directed to the corresponding author/s.
