# Supplementary material for: Inflammatory and immune pathways in Ménière’s disease: a multi-database bibliometric and visualized analysis of research trends
Source: Front Cell Infect Microbiol. 2026 Sep 29;16:1951199. doi: 10.3389/fcimb.2026.1951199 (PMC13657947; doi:10.3389/fcimb.2026.1951199)
Supplement: Supplementary file 1 [file Table1.docx]

**Supplementary Table 1.** **Details of retrieval strategies for each database.**

| **Database** | **Search Strategy** | **Time Span** | **Language** | **Document Type** | **Initial Records Retrieved** |
| --- | --- | --- | --- | --- | --- |
| Web of Science Core Collection (WOSCC) | TS =(("Meniere disease" OR "Meniere's disease" OR "Ménière disease" OR "Ménière's disease" OR "Meniere syndrome" OR "Ménière syndrome" OR "endolymphatic hydrops" OR "cochlear hydrops" OR "vestibular hydrops") AND ("immun*" OR "inflamm*" OR "immune response*" OR "immune mechanism*" OR "immune-mediated" OR "autoimmun*" OR "autoinflamm*" OR "allerg*" OR "hypersensitiv*" OR "atopy" OR "atopic" OR "IgE" OR "immunoglobulin E" OR "cytokine*" OR "chemokine*" OR "interleukin*" OR "IL-1" OR "IL-4" OR "IL-5" OR "IL-6" OR "IL-10" OR "IL-13" OR "TNF" OR "tumor necrosis factor" OR "NLRP3" OR "inflammasome" OR "pyroptosis" OR "eosinophil*" OR "mast cell*" OR "histamine" OR "leukotriene*" OR "Th1" OR "Th2" OR "type 2 inflammation" OR "type 2 immunity" OR "immune phenotype*" OR "endotype*" OR "biomarker*" OR "immunotherap*" OR "therapeutic target*")) | Jan 1, 2006 – Dec 31, 2025 | English | Article, Review | 422 |
| Scopus | TITLE-ABS-KEY(("Meniere disease" OR "Meniere's disease" OR "Ménière disease" OR "Ménière's disease" OR "endolymphatic hydrops") AND (immun* OR inflamm* OR autoimmun* OR autoinflamm* OR allerg* OR hypersensitiv* OR IgE OR cytokine* OR chemokine* OR interleukin* OR NLRP3 OR inflammasome OR pyroptosis OR eosinophil* OR "mast cell*" OR Th2 OR "type 2 inflammation" OR "immune phenotype*" OR endotype* OR biomarker* OR immunotherap*)) | Jan 1, 2006 – Dec 31, 2025 | English | Article, Review | 804 |

**Supplementary Table 2. Temporal trends in publication output of the top 10 authors.**

year indicates the publication year; freq represents the number of publications by each author in that year; TC denotes total citations received by the publications; and TCpY refers to citations per year, calculated as total citations divided by the number of years since publication.

| **Author** | **year** | **freq** | **TC** | **TCpY** |
| --- | --- | --- | --- | --- |
| ARAN, ISMAEL | 2011 | 2 | 168 | 10.5 |
| ARAN, ISMAEL | 2012 | 1 | 34 | 2.267 |
| ARAN, ISMAEL | 2013 | 2 | 80 | 5.714 |
| ARAN, ISMAEL | 2014 | 1 | 34 | 2.615 |
| ARAN, ISMAEL | 2016 | 1 | 119 | 10.818 |
| ARAN, ISMAEL | 2017 | 1 | 61 | 6.1 |
| ARAN, ISMAEL | 2018 | 1 | 86 | 9.556 |
| ARAN, ISMAEL | 2019 | 1 | 81 | 10.125 |
| ARAN, ISMAEL | 2021 | 3 | 84 | 14 |
| ARAN, ISMAEL | 2025 | 2 | 5 | 2.5 |
| FREJO, LIDIA | 2016 | 1 | 119 | 10.818 |
| FREJO, LIDIA | 2017 | 1 | 61 | 6.1 |
| FREJO, LIDIA | 2018 | 1 | 86 | 9.556 |
| FREJO, LIDIA | 2019 | 1 | 81 | 10.125 |
| FREJO, LIDIA | 2021 | 1 | 28 | 4.667 |
| FREJO, LIDIA | 2022 | 1 | 61 | 12.2 |
| FREJO, LIDIA | 2023 | 2 | 48 | 12 |
| FREJO, LIDIA | 2024 | 2 | 36 | 12 |
| FREJO, LIDIA | 2025 | 2 | 18 | 9 |
| GALLEGO-MARTINEZ, ALVARO | 2017 | 1 | 61 | 6.1 |
| GALLEGO-MARTINEZ, ALVARO | 2018 | 2 | 116 | 12.889 |
| GALLEGO-MARTINEZ, ALVARO | 2019 | 1 | 81 | 10.125 |
| GALLEGO-MARTINEZ, ALVARO | 2020 | 2 | 50 | 7.143 |
| GALLEGO-MARTINEZ, ALVARO | 2021 | 3 | 85 | 14.167 |
| GALLEGO-MARTINEZ, ALVARO | 2024 | 1 | 16 | 5.333 |
| GALLEGO-MARTINEZ, ALVARO | 2025 | 2 | 5 | 2.5 |
| HOA, MICHAEL | 2015 | 1 | 46 | 3.833 |
| HOA, MICHAEL | 2017 | 1 | 143 | 14.3 |
| HOA, MICHAEL | 2019 | 1 | 8 | 1 |
| HOA, MICHAEL | 2021 | 2 | 34 | 5.667 |
| HOA, MICHAEL | 2022 | 2 | 17 | 3.4 |
| HOA, MICHAEL | 2023 | 3 | 33 | 8.25 |
| HOA, MICHAEL | 2024 | 1 | 3 | 1 |
| HOA, MICHAEL | 2025 | 1 | 7 | 3.5 |
| ISHIYAMA, AKIRA | 2006 | 1 | 40 | 1.905 |
| ISHIYAMA, AKIRA | 2010 | 2 | 47 | 2.765 |
| ISHIYAMA, AKIRA | 2011 | 1 | 17 | 1.063 |
| ISHIYAMA, AKIRA | 2012 | 1 | 29 | 1.933 |
| ISHIYAMA, AKIRA | 2013 | 2 | 39 | 2.786 |
| ISHIYAMA, AKIRA | 2018 | 1 | 36 | 4 |
| ISHIYAMA, AKIRA | 2019 | 3 | 27 | 3.375 |
| ISHIYAMA, AKIRA | 2020 | 1 | 5 | 0.714 |
| ISHIYAMA, AKIRA | 2021 | 1 | 15 | 2.5 |
| ISHIYAMA, AKIRA | 2022 | 1 | 54 | 10.8 |
| ISHIYAMA, AKIRA | 2023 | 4 | 12 | 3 |
| ISHIYAMA, AKIRA | 2024 | 1 | 7 | 2.333 |
| ISHIYAMA, AKIRA | 2025 | 1 | 2 | 1 |
| ISHIYAMA, GAIL | 2006 | 1 | 40 | 1.905 |
| ISHIYAMA, GAIL | 2010 | 1 | 30 | 1.765 |
| ISHIYAMA, GAIL | 2011 | 1 | 17 | 1.063 |
| ISHIYAMA, GAIL | 2012 | 1 | 29 | 1.933 |
| ISHIYAMA, GAIL | 2013 | 2 | 39 | 2.786 |
| ISHIYAMA, GAIL | 2018 | 1 | 36 | 4 |
| ISHIYAMA, GAIL | 2019 | 2 | 19 | 2.375 |
| ISHIYAMA, GAIL | 2021 | 2 | 19 | 3.167 |
| ISHIYAMA, GAIL | 2022 | 1 | 54 | 10.8 |
| ISHIYAMA, GAIL | 2023 | 3 | 8 | 2 |
| ISHIYAMA, GAIL | 2024 | 1 | 7 | 2.333 |
| ISHIYAMA, GAIL | 2025 | 1 | 2 | 1 |
| LOPEZ, IVAN A. | 2010 | 2 | 47 | 2.765 |
| LOPEZ, IVAN A. | 2012 | 1 | 29 | 1.933 |
| LOPEZ, IVAN A. | 2013 | 2 | 39 | 2.786 |
| LOPEZ, IVAN A. | 2018 | 1 | 36 | 4 |
| LOPEZ, IVAN A. | 2019 | 2 | 19 | 2.375 |
| LOPEZ, IVAN A. | 2020 | 1 | 5 | 0.714 |
| LOPEZ, IVAN A. | 2021 | 1 | 15 | 2.5 |
| LOPEZ, IVAN A. | 2023 | 2 | 5 | 1.25 |
| LOPEZ, IVAN A. | 2025 | 1 | 2 | 1 |
| LOPEZ-ESCAMEZ, JOSE A. | 2010 | 1 | 47 | 2.765 |
| LOPEZ-ESCAMEZ, JOSE A. | 2011 | 2 | 168 | 10.5 |
| LOPEZ-ESCAMEZ, JOSE A. | 2013 | 2 | 80 | 5.714 |
| LOPEZ-ESCAMEZ, JOSE A. | 2014 | 1 | 34 | 2.615 |
| LOPEZ-ESCAMEZ, JOSE A. | 2015 | 2 | 241 | 20.083 |
| LOPEZ-ESCAMEZ, JOSE A. | 2016 | 2 | 162 | 14.727 |
| LOPEZ-ESCAMEZ, JOSE A. | 2017 | 1 | 61 | 6.1 |
| LOPEZ-ESCAMEZ, JOSE A. | 2020 | 3 | 93 | 13.286 |
| LOPEZ-ESCAMEZ, JOSE A. | 2021 | 1 | 28 | 4.667 |
| LOPEZ-ESCAMEZ, JOSE A. | 2023 | 2 | 48 | 12 |
| LOPEZ-ESCAMEZ, JOSE A. | 2024 | 3 | 60 | 20 |
| LOPEZ-ESCAMEZ, JOSE A. | 2025 | 5 | 9 | 4.5 |
| SOTO-VARELA, ANDRES | 2011 | 2 | 168 | 10.5 |
| SOTO-VARELA, ANDRES | 2012 | 1 | 34 | 2.267 |
| SOTO-VARELA, ANDRES | 2013 | 2 | 80 | 5.714 |
| SOTO-VARELA, ANDRES | 2014 | 1 | 34 | 2.615 |
| SOTO-VARELA, ANDRES | 2016 | 1 | 119 | 10.818 |
| SOTO-VARELA, ANDRES | 2017 | 1 | 61 | 6.1 |
| SOTO-VARELA, ANDRES | 2018 | 1 | 86 | 9.556 |
| SOTO-VARELA, ANDRES | 2019 | 1 | 81 | 10.125 |
| SOTO-VARELA, ANDRES | 2021 | 3 | 84 | 14 |
| SOTO-VARELA, ANDRES | 2023 | 1 | 20 | 5 |
| SOTO-VARELA, ANDRES | 2025 | 2 | 6 | 3 |
| STRUPP, MICHAEL | 2006 | 1 | 38 | 1.81 |
| STRUPP, MICHAEL | 2007 | 1 | 245 | 12.25 |
| STRUPP, MICHAEL | 2011 | 2 | 168 | 10.5 |
| STRUPP, MICHAEL | 2012 | 1 | 59 | 3.933 |
| STRUPP, MICHAEL | 2013 | 1 | 38 | 2.714 |
| STRUPP, MICHAEL | 2014 | 1 | 26 | 2 |
| STRUPP, MICHAEL | 2015 | 2 | 55 | 4.583 |
| STRUPP, MICHAEL | 2017 | 2 | 38 | 3.8 |
| STRUPP, MICHAEL | 2019 | 1 | 22 | 2.75 |
| STRUPP, MICHAEL | 2021 | 1 | 15 | 2.5 |
| STRUPP, MICHAEL | 2022 | 1 | 24 | 4.8 |
| STRUPP, MICHAEL | 2023 | 2 | 13 | 3.25 |

**Supplementary Figure 1. Dual-map overlay of citation flows across disciplines.** Citation pathways link journals in “molecular biology, immunology,” “molecular biology, genetics,” and “dentistry, dermatology, surgery,” showing inter-field citation relationships.
